# The effect of self-efficacy on feedback seeking among undergraduate students: the role of perceived risk cost and student-LMX

**DOI:** 10.3389/fpsyg.2026.1847026

**Published:** 2026-08-04

**Authors:** Han Xie, Li Cheng

**Affiliations:** 1 School of Educational Sciences, Hubei University of Education, Wuhan, China; 2 Hubei Teacher Education Research Center, Wuhan, China

**Keywords:** feedback seeking behavior, perceived benefit, perceived risk, self-efficacy, student-LMX

## Abstract

Drawing on social cognitive theory and the cost-value framework, this study examines the mediating roles of perceived benefit and perceived risk in the relationship between self-efficacy and feedback seeking, and the moderating roles of student-LMX and gender, among 502 Chinese undergraduates. Results from structural equation modeling and multi-group analysis revealed that self-efficacy positively predicted perceived benefit and negatively predicted perceived risk, which in turn influenced both direct and indirect feedback seeking. LMX moderated the risk pathway, making it significant only under high-LMX conditions, whereas the benefit pathway remained stable across LMX levels; gender showed no moderating effect. These findings clarify the dual mechanisms through which self-efficacy translates into feedback seeking, advance LMX theory by demonstrating its differential moderation, and extend feedback seeking research to educational settings with a culturally grounded interpretation accounting for face concerns in Chinese higher education. Practically, educators should not only build high-quality relationships with students but also cultivate a feedback culture that normalizes seeking feedback as a growth-oriented practice rather than a sign of inadequacy.

## Introduction

Feedback refers to information provided by others regarding their perceptions of one's performance (Hattie and Timperley, 2007). Through understanding, analyzing, and internalizing feedback from others, learners can gain a clearer awareness of their strengths and weaknesses (Ashford and Blatt, 2003). In this sense, the process of receiving and acting upon feedback is also a process of learning (Evans, 2013). Although feedback seeking has been widely studied in workplace contexts (Joughin et al., 2021), Leenknecht and Carless (2023) identified a research gap on this topic in their scoping review of feedback seeking among students in higher education. Undergraduate students may receive feedback from diverse sources and in various forms—either directly through explicit solicitation, or indirectly by inferring from informal environmental cues (Ashford and Cummings, 1983; Rae and Cochrane, 2008; Sun, 2021). Although teachers represent the primary source of feedback in educational settings (Ames and Lau, 1982; Ghbari et al., 2014), spontaneous feedback from this social source is often limited, as instructors are not always able to provide timely and individualized feedback to every student (Miao et al., 2006; Bergh et al., 2013). This reality implies that active feedback seeking may be essential for students to obtain timely and useful information regarding their academic performance. Indeed, as a key self-regulated learning strategy, feedback seeking is of significant value to individual learning and development (Ashford and Tsui, 1991; Anseel and Lievens, 2010). Beyond the question of why students seek feedback, Leenknecht and Carless (2023) also pointed to the need to examine the diversity of drivers and to adopt integrative approaches. One important yet underexplored driver is the relational context in which feedback seeking occurs (Leenknecht et al., 2019). Drawing on LMX theory, we propose that student-LMX serves as a key boundary condition that moderates the effects of self-efficacy and cost-value perceptions on feedback seeking, thereby addressing the call for contextualized and integrative research on feedback seeking in higher education.

Self-efficacy has important implications for feedback seeking behaviors (Ashford and Cummings, 1983; VandeWalle and Cummings, 1997). This study is guided by social cognitive theory and the cost-value framework to delineate the cognitive pathways through which self-efficacy influences students' feedback seeking in higher education (VandeWalle et al., 2000; Vandewalle, 2004). Our work thus facilitates an empirical understanding of the mediating roles of perceived risk and perceived benefit underlying students' perceptions and behavioral decisions regarding feedback seeking, distinguishing between direct and indirect forms of feedback seeking. Furthermore, we identify two key moderating conditions that may shape the strength of these relationships: student-LMX, representing the relational context, and gender, representing an individual demographic characteristic. High-quality LMX is expected to amplify the positive influence of self-efficacy and perceived benefit while buffering the inhibiting effects of perceived risk, whereas gender is examined as a potential boundary condition given the mixed findings in the feedback literature regarding gender differences. By integrating these mediating and moderating mechanisms within a single analytical framework, this study offers a more comprehensive account of when and how self-efficacy translates into different types of feedback seeking.

To test the proposed model, we collected survey data and used structural equation modeling to examine the hypothesized relationships, including the indirect effects of self-efficacy through perceived risk and perceived benefit, as well as the moderating roles of student-LMX and gender.

### Self-efficacy and feedback seeking behavior

Self-efficacy—defined as individuals' beliefs in their capability to succeed in specific situations or accomplish specific tasks (Bandura, 1986)—has long been considered a central premise in the feedback seeking literature (Chen and Zhang, 2019; Crommelinck, 2013). The theoretical rationale for this expectation is that self-efficacy reduces the threat inherent in receiving feedback, as individuals with high self-efficacy possess less fragile egos and are therefore more willing to solicit diagnostic information about their performance Renn and Fedor (2001). Empirical evidence has generally supported this positive association. A meta-analysis by (Anseel et al. 2015) confirmed that self-efficacy is positively related to feedback seeking behavior. In educational research, academic self-efficacy is significantly positively correlated with students' feedback-seeking behavior (Fatima et al., 2022). (Gan et al. 2021) found that English language self-efficacy significantly influenced both feedback behavior and preference among Chinese university students.

However, recent research has complicated this straightforward picture. (Sherf and Morrison 2020) found that high self-efficacy may reduce feedback seeking by lowering perceived feedback value, and that this effect depends on perspective taking. The relationship between self-efficacy and feedback seeking is further complicated when differentiating between the two primary strategies of feedback seeking: feedback inquiry—directly asking others for feedback on one's performance—and feedback monitoring—actively and strategically attending to feedback cues present in the environment (Ashford and Cummings, 1983; Brett et al., 1990). Xu and Wang (2024) examined this distinction in the context of second language writing with a sample of 606 Chinese participants. Their quantitative results revealed that linguistic self-efficacy negatively impacted feedback inquiry, whereas performance self-efficacy positively impacted it; self-efficacy positively influenced both feedback monitoring and inquiry. This suggests that not all forms of self-efficacy operate uniformly on feedback seeking, and the effect may differ depending on the specific strategy employed.

To clarify this issue, we propose the following hypotheses:

H1a: Self-efficacy is positively associated with direct feedback seeking (feedback inquiry).
H1b: Self-efficacy is positively associated with indirect feedback seeking (feedback monitoring).

Consistent with the dominant theoretical position, we expect self-efficacy to promote both forms of feedback seeking. However, given the distinct nature of the two strategies—with inquiry involving higher social risk and self-exposure than monitoring—we also explore whether the strength of the relationship differs across the two pathways.

### Cost–value perception

According to Bandura (1986, 1982); Bandura and Wood (1989), self-efficacy operates through four main processes: cognitive, motivational, affective, and selection processes. Of particular relevance to feedback seeking is the affective process, through which self-efficacy shapes individuals' emotional responses to perceived threats and challenges—including the anxiety, fear, or discomfort associated with exposing one's shortcomings to others (Bandura, 1986; Gino et al., 2012). Individuals with high self-efficacy are more likely to approach challenging situations with confidence rather than apprehension, whereas those with low self-efficacy tend to magnify potential risks and anticipate negative outcomes. Applied to the feedback context, this theoretical logic suggests that self-efficacy should fundamentally shape how students appraise both the benefits and the risks of seeking feedback.

The cost-value framework provides a useful lens for understanding how self-efficacy translates into feedback seeking behavior. Based on this framework, individuals are assumed to engage in a cognitive calculus in which they weigh the anticipated benefits of seeking feedback against its perceived costs before deciding whether to initiate feedback seeking (Anseel et al., 2015; Morrison and Vancouver, 2000; Lee, 2002; Vandewalle, 2004). Extending this logic, we argue that self-efficacy functions as a key personal resource that systematically biases this cost-value calculus in a favorable direction. Specifically, high self-efficacy should simultaneously amplify the perceived value of feedback—by enhancing individuals' confidence in their ability to process and utilize feedback effectively—while attenuating its perceived risks—by reducing fears of negative evaluation, embarrassment, and loss of face (Ashford, 1986; Ashford and Blatt, 2003; Brown et al., 2001). Conversely, individuals with low self-efficacy are more sensitive to negative evaluation, which can threaten their self-worth and inhibit proactive behaviors (Anseel et al., 2015; Weiss and Knight, 1980; William, 1987). This dual-pathway logic positions self-efficacy as a critical antecedent that shapes feedback seeking through both value-enhancing and risk-reducing mechanisms.

Recent empirical studies conducted in higher education contexts have provided preliminary support for the cost-value framework (Zhang, 2025; Gu and Man, 2026; Wu et al., 2019), generally suggesting that value perceptions are positively associated with feedback seeking (Podsakoff and Farh, 1989), whereas cost perceptions tend to function as a barrier (Spooner et al., 2024; Young and Carless, 2024). However, the simultaneous mediating roles of perceived benefit and perceived risk cost in the relationship between self-efficacy and feedback seeking remain unexplored, particularly when distinguishing between direct and indirect feedback seeking strategies (Ashford and Cummings, 1983). This gap warrants attention, as the two strategies entail different levels of social exposure and psychological risk: direct feedback seeking, explicitly asking others for feedback—involves higher social risk and self-exposure, whereas indirect feedback seeking, may be less susceptible to such social costs (Ashford and Cummings, 1983). It thus remains to be determined whether self-efficacy exerts its effects on these two strategies through the same cognitive pathways, or whether the mediating mechanisms differ in strength or significance.

Given the above theoretical rationale, we propose the following hypotheses:

H2a: Perceived benefit mediates the positive relationship between self-efficacy and direct feedback seeking.
H2b: Perceived benefit mediates the positive relationship between self-efficacy and indirect feedback seeking.
H3a: Perceived risk mediates the negative relationship between self-efficacy and direct feedback seeking.
H3b: Perceived risk mediates the negative relationship between self-efficacy and indirect feedback seeking.

### The moderating role of gender

Gender has been increasingly recognized as a potential moderator in feedback-related processes, though empirical findings remain mixed. In organizational settings, Lam et al. (2015) found that newcomers “negative feedback seeking positively predicted supervisors” liking of female newcomers, whereas this effect was not observed for male newcomers, suggesting that gender shapes how feedback-seeking behaviors are socially interpreted. In educational contexts, (Yang et al. 2025) found that male students exhibited stronger predictive power of process feedback over feedback self-efficacy, whereas female students with higher feedback self-efficacy achieved greater academic success. Gender differences have also been observed in peer feedback contexts, with female students demonstrating a stronger preference for and providing higher-quality feedback compared to their male counterparts. Gender role theory suggests that men are expected to demonstrate agentic traits such as independence and self-reliance, whereas women are expected to display communal traits such as warmth and nurturance (Eagly and Karau, 2002). Since feedback seeking entails admitting incompetence and dependence on others—behaviors that deviate from agentic role expectations (Rosenthal and Jacobson, 1968)—men may perceive higher social costs when seeking feedback compared to women (Miller and Karakowsky, 2005). We therefore propose:

H4: Gender moderates the indirect effects of self-efficacy on (a) direct feedback seeking and (b) indirect feedback seeking through perceived benefit and perceived risk.

### The moderating role of student-LMX

LMX theory seeks to explain the how leaders are able to direct the behavior of their subordinates (Cropanzano and Mitchell, 2005; Dienesch and Liden, 1986; Gerstner and Day, 1997; Liden et al., 2006; Sparrowe and Liden, 1997). LMX is a central consideration in the deployment of a learner-centered pedagogy within universities (Farr-Wharton et al., 2018). Student-LMX refers to the relationship formed between students and their lecturers through learning interactions and teaching activities. LMX is applicable to the lecturer-student dynamic wherein lecturers direct student's learning behavior through the relationships that they form (Farr-Wharton et al., 2018). A teacher may indicate the capacity for a closer relationship with their students. When students reciprocate, a mutually beneficial relationship develops over time (Cropanzano and Mitchell, 2005). Through reciprocity, the ongoing process of give and take, the students benefit as a result of having enhanced access to the resources and the attention of their teacher (Mosley et al., 2014). Once the relationship between supervisors and subordinates is clearly recognized, it can effectively predict an individual's behavior and attitude. High-quality LMX can provide individuals with more psychological resources such as social support and collective cohesion (Fairhurst and Chandler, 1989; Hsiung and Tsai, 2009). Positive dyadic exchanges or higher quality connections between lecturers and students should be related to greater engagement in the learning process (Atwater and Carmeli, 2009; Jacques et al., 2012).

In the Chinese cultural context, feedback seeking is particularly challenging due to face concerns and the influence of Confucian values. Hwang et al. (2002) demonstrated that face-related cultural attitudes such as the fear of losing face, significantly shape students' feedback seeking behaviors, as admitting incompetence or dependence in public is perceived as a direct threat to one's social image. Chen et al. (2023) further found that perceived face threat mediates the relationship between negative leadership and employee feedback seeking, suggesting that face concerns function as a key psychological mechanism that inhibits proactive feedback behaviors. High-quality LMX helps students “save face” when exposing their inadequacies, thereby buffering the social costs inherent in feedback seeking and activating the risk pathway. Lan et al. (2020) found that LMX incongruence decreases employees' psychological safety, which in turn undermines feedback seeking. In contrast, perceived benefit is more cognitively driven, the judgment that feedback is useful for performance improvement is less contingent on relational quality or face concerns, and thus remains relatively stable across LMX levels. We therefore propose:

H5a: LMX moderates the risk-based pathway, such that the indirect effects are significant only when LMX is high.
H5b: LMX does not moderate the benefit-based pathway, such that the indirect effects remain stable across LMX levels.

### The present study

This study aims to examine the influencing mechanism of self-efficacy by addressing the following research questions:

RQ1: how is undergraduate's self-efficacy related to perceived feedback risk (PR) and perceived feedback benefit (PB)?
RQ2: how is undergraduate's self-efficacy associated with the frequency of direct feedback seeking (DFS) and indirect feedback seeking (IFS) in feedback acquisition processes?
RQ3: whether gender and Student-LMX exert moderating effects on the proposed relationships in this model.

**Figure 1:**
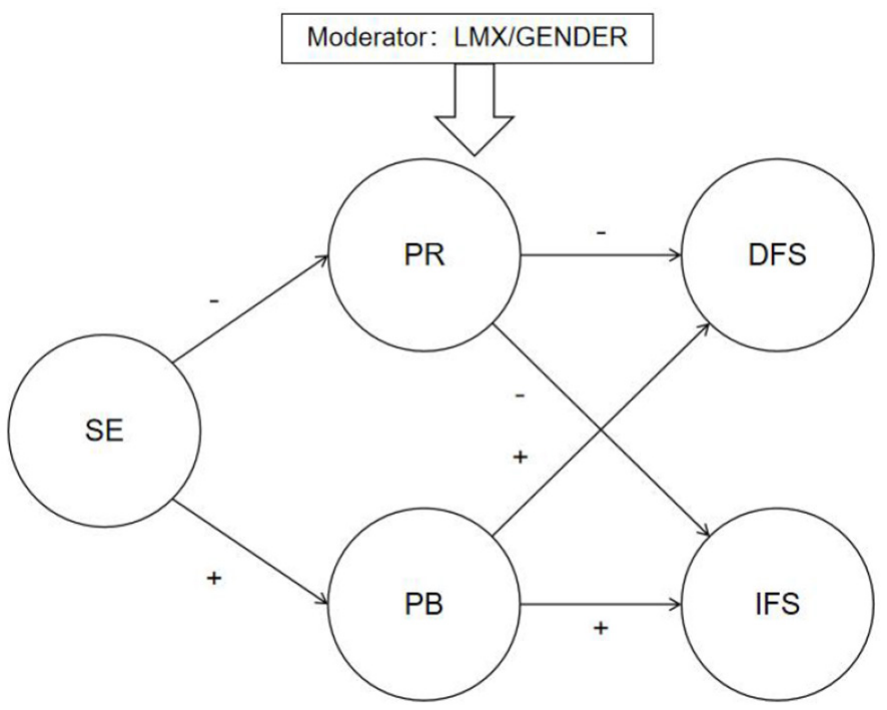
Theoretical model.

Based on the literature, we hypothesis that students' self-efficacy is related to the perceived feedback resistance or perceived feedback benefit, which in turn are associated with the direct or indirect feedback seeking frequency. Additionally, gender and Student-LMX will moderate the core paths of this structural model, as depicted in the corresponding structural model in Figure 1.

## Method

### Participants

This survey was conducted in June 2026 across three provinces in China—Jiangsu, Henan, and Hubei. A total of 528 questionnaires were distributed, and after excluding invalid responses (e.g., duplicate submissions and excessively short or long completion times), 502 valid responses were retained, yielding an effective response rate of 95.1%. Participants were told that their participation was entirely voluntary. Upon consenting, they were given 15 min to complete the questionnaire and were assured of their right to withdraw at any time. Among the final sample, 161 (32.1%) were male and 341 (67.9%) were female. The mean age of the sample was 19.70 years (SD = 0.97, ranging from 17 to 23).

### Measures

#### Self-efficacy

Self-efficacy was assessed using eight items drawn from the self-efficacy sub-scale of the Motivated Strategies for Learning Questionnaire (MSLQ), originally developed by Pintrich (1991). The items capture students' self-perceptions of their academic capabilities (e.g., “I am confident that I can understand the most complex material presented in this course”). Each item was rated on a seven-point scale ranging from one (strongly disagree) to seven (strongly agree). Responses were averaged, with higher scores indicating stronger self-efficacy. In the present study, Cronbach's α was 0.915.

#### Feedback seeking

Feedback seeking was measured using the Feedback Seeking Behavior Scale (FSB), originally developed by Ashford (1986) in the workplace context. In this study, the scale was adapted for use in the educational setting, with some items modified for clarity to capture the frequency of students' feedback-seeking behaviors. The scale consists of two subscales: direct feedback seeking (three items) and indirect feedback seeking (four items), each rated on a five-point scale ranging from one (very infrequently) to five (very frequently). Responses were averaged, with higher scores indicating more frequent feedback seeking. The measure demonstrated good reliability in the present study: Cronbach's α for the direct feedback seeking sub-scale was 0.897, for the indirect feedback seeking sub-scale was 0.928, and for the overall scale was 0.946.

#### Perceived risk

Students' feedback-seeking cost perceptions were measured using three items drawn from the scale originally developed by Ashford (1986) in the workplace context. In this study, the items were adapted for the educational setting to capture students' perceptions of potential risks associated with seeking feedback (e.g., “I think my teacher would think worse of me if I asked him/her for feedback”). Each item was rated on a five-point scale ranging from one (strongly disagree) to five (strongly agree). Responses were averaged, with higher scores indicating higher perceived risk. The measure demonstrated acceptable reliability in the present study, Cronbach's α = 0.785.

#### Perceived value

Perceived feedback value was measured using three items adapted from Keeping and Levy (2000) perceived utility scale. The items were slightly modified to fit the educational context, capturing students' perceptions of the usefulness of feedback from their instructors. A sample item is: “Feedback from my teacher will help me do my course work better.” Participants rated each item on a five-point scale ranging from one (strongly disagree) to five (strongly agree). Responses were averaged, with higher scores indicating greater perceived value of feedback. In the present study, Cronbach's α for this scale was 0.977, indicating good reliability.

#### Student-LMX

Student-LMX was adapted by LMX (Leader-Member Exchange Scale), a scale originally developed by (Graen and Uhl-Bien, 1995). The scale contains seven statements about students' feelings about their teacher (e.g. “I trust my teacher and support him in all his plans and decisions related to scientific research.”). Items were responded to on a five-point scale ranging from one (strongly disagree) to five (strongly agree). Responses were averaged with the scores varying between one and five. Higher scores indicate higher quality of teacher-student relationship. The scale has been used extensively in Chinese samples (e.g., Zhao et al., 2019) with good reliability (α = 0.91) to represent interaction relationships between supervisor and followers in China. In this study, the Cronbach's α for the scale was 0.960.

## Results

### Common method bias

To assess the potential threat of common method bias, we conducted a common latent factor (CLF) test by comparing a baseline model (without CLF) with a CLF-adjusted model (with CLF loadings constrained to be equal). The chi-square difference between the two models was not significant (Δχ^2^ (1) = 3.066, *p* = 0.080), indicating that common method variance did not substantially influence the findings of this study.

### Validity assessment

To assess discriminant validity, we conducted confirmatory factor analysis (CFA) and compared the hypothesized six-factor model with several alternative models. As shown in Table 1, the six-factor model yielded a good fit to the data (χ^2^/df = 4.39, CFI = 0.93, TLI = 0.92, RMSEA = 0.08), and consistently outperformed all alternative models in which two or more constructs were merged. For instance, combining SE, PR, and PB into one factor led to a significant deterioration in model fit (CFI = 0.91), and further collapsing DFS and IFS into a single factor also resulted in poorer fit. The more parsimonious models showed progressively worse fit, with CFI values ranging from 0.86 to 0.75. These results provide strong support for the discriminant validity of the six constructs.

**Table 1:** Confirmatory factor analysis (CFA).

Model	χ^2^	df	χ^2^/df	CFI	TLI	RMSEA
6-factor model	1,470.88	335	4.39	0.93	0.92	0.08
5-factor model	1,754.75	340	5.16	0.91	0.90	0.09
4-factor model	1,748.30	344	5.37	0.91	0.90	0.09
3-factor model	2,630.30	347	7.58	0.86	0.84	0.12
2-factor model	3,961.17	349	11.35	0.77	0.75	0.14
1-factor model	4,273.61	350	12.21	0.75	0.73	0.15

### Descriptive statistics

Table 2 presents the means, standard deviations, and correlations among all variables. Pearson correlations indicated that all constructs were significantly correlated in the expected directions (*p* < 0.01 for all coefficients). Self-efficacy (SE) was positively associated with perceived benefit (PB, *r* = 0.604), feedback seeking (DFS, *r* = 0.626; IFS, *r* = 0.625), and LMX (*r* = 0.642), and negatively associated with perceived risk (PR, *r* = −0.592). Perceived risk was negatively correlated with PB, DFS, IFS, and LMX, whereas perceived benefit showed strong positive correlations with DFS (*r* = 0.866), IFS (*r* = 0.815), and LMX (*r* = 0.804). These patterns provide preliminary support for the hypothesized relationships.

**Table 2:** Descriptive statistics and correlation analysis.

Variables	M	SD	SE	PR	PB	DFS	IFS	LMX
SE	5.76	0.96	1					
PR	3.15	1.26	−0.592^**^	1				
PB	5.38	1.28	0.604^**^	−0.684^**^	1			
DFS	4.68	1.13	0.626^**^	−0.773^**^	0.866^**^	1		
IFS	3.74	0.81	0.625^**^	−0.773^**^	0.815^**^	0.879^**^	1	
LMX	3.72	0.92	0.642^**^	−0.752^**^	0.804^**^	0.816^**^	0.800^**^	1

*N* = 502; ^**^p < 0.01.

### Structural model

The structural path analysis was conducted to test the hypothesized relationships among the constructs. The overall fit of the structural model was evaluated using multiple fit indices. The structural model yielded a reasonable fit to the data: χ^2^ (181) = 827.88, χ^2^/df = 4.57, CFI = 0.94, TLI = 0.93, NFI = 0.93, IFI = 0.94, GFI = 0.85, AGFI = 0.80, RMSEA = 0.08. According to Hu and Bentler (1999); Browne and Cudeck (1993), and Byrne (2010); Hayes and Preacher (2013), all fit indices met or exceeded their respective commonly acceptable levels, indicating that the structural model represents an acceptable fit to the data and supports the validity of the hypothesized relationships. As shown in Table 3, all proposed paths were statistically significant at the *p* < 0.001 level. Self-efficacy (PSE) had a strong positive effect on perceived benefit (PB, β = 0.826, SE = 0.055, CR = 14.973) and a strong negative effect on perceived risk (PR, β = −0.949, SE = 0.062, CR = −15.228). In turn, perceived benefit significantly and positively predicted both direct feedback seeking (DFS, β = 0.501, SE = 0.055, CR = 9.057) and indirect feedback seeking (IFS, β = 0.230, SE = 0.042, CR = 5.527). Conversely, perceived risk exerted significant negative influences on both direct feedback seeking (β = −0.437, SE = 0.057, CR = −7.610) and indirect feedback seeking (β = −0.365, SE = 0.044, CR = −8.309). These results indicate that self-efficacy enhances the perceived benefits while reducing perceived risks, and both benefit and risk perceptions subsequently shape feedback-seeking behaviors—with benefit exerting a stronger impact on direct seeking and risk exerting comparable negative effects on both types of seeking.

**Table 3:** Hypothesis testing results.

Path	Estimate	Std. error	Critical ratio	*p*-value
SE → PB	0.826	0.055	14.973	0.000^***^
SE → PR	−0.949	0.062	−15.228	0.000^***^
PB → IFS	0.230	0.042	5.527	0.000^***^
PR → DFS	−0.437	0.057	−7.610	0.000^***^
PB → DFS	0.501	0.055	9.057	0.000^***^
PR → IFS	−0.365	0.044	−8.309	0.000^***^

SE, self-efficacy; PR, perceived risk; PB, perceived benefit; DFS, direct feedback seeking; IFS, indirect feedback seeking; LMX, leader-member exchange. ^***^ *p* < 0.001.

### Moderating effect of gender

To examine the moderating effect (Table 4), multiple group analysis (MGA) with AMOS 18 was performed following the procedure recommended by Jöreskog and Sörbom (1993). Specifically, the data set was first split into two gender groups (male and female). Next, a structural equation model (SEM) was estimated with equality constraints imposed on all paths, fixing each regression weight to be equal across the two groups. Then, a second SEM model was estimated without any equality constraints, allowing all path coefficients to be freely estimated. The significance of moderating effects is determined based on the chi-square difference test whereby a critical value >3.841 at the 5% level is considered adequate for a significant moderating effect to occur.

**Table 4:** Gender moderating effects.

Path	Male	Female	Chi-square difference	*p*-value
SE → PB	0.769^***^	0.858^***^	0.604	0.437
PB → IFS	0.374^***^	0.214^***^	3.197	0.074
PB → DFS	0.597^***^	0.503^***^	0.002	0.964
SE → PR	−0.934^***^	−0.900^***^	0.072	0.788
PR → IFS	−0.224^**^	−0.396^***^	2.877	0.090
PR → DFS	−0.403^***^	−0.409^***^	0.636	0.425

SE, self-efficacy; PR, perceived risk; PB, perceived benefit; DFS, direct feedback seeking; IFS, indirect feedback seeking.

^***^p < 0.001, ^**^p < 0.01.

As shown in Table 3, all paths between male and female groups were non-significant, as indicated by the chi-square difference tests (all Δχ^2^ < 3.841, all *p* > 0.05). Specifically, the chi-square differences for the paths from SE to PB (Δχ^2^ = 0.604, *p* = 0.437), PB to IFS (Δχ^2^ = 3.197, *p* = 0.074), PB to DFS (Δχ^2^ = 0.002, *p* = 0.964), SE to PR (Δχ^2^ = 0.072, *p* = 0.788), PR to IFS (Δχ^2^ = 2.877, *p* = 0.090), and PR to DFS (Δχ^2^ = 0.636, *p* = 0.425) all fell below the critical threshold, suggesting no significant gender differences in any of the hypothesized relationships. Therefore, we conclude that gender does not moderate the relationships between self-efficacy, perceived risk, perceived benefit, and feedback-seeking behaviors. A possible justification is that the educational context in which participants operate provides equal opportunities and support for both genders to develop relevant capabilities and engage in feedback-seeking activities, thereby attenuating potential gender-based differences.

### Moderating effect of student-LMX

Following the same multiple group analysis procedure as for gender, we split the sample into high and low LMX groups (top and bottom 33%).

As shown in Table 5, the chi-square difference tests revealed that LMX significantly moderated three of the six paths: SE → PR (Δχ^2^ = 26.633, *p* < 0.001), PR → IFS (Δχ^2^ = 5.388, *p* = 0.020), and SE → PB (Δχ^2^ = 4.721, *p* = 0.030). However, no significant moderating effects were found for PR → DFS (Δχ^2^ = 2.563, *p* = 0.109), PB → DFS (Δχ^2^ = 0.019, *p* = 0.890), or PB → IFS (Δχ^2^ = 0.251, *p* = 0.617). These findings indicate that LMX partially moderates the relationships among the constructs, primarily through the risk-related pathways. A possible justification is that high-quality LMX provides greater trust (Brown and Dutton, 1995), support, and psychological safety, thereby amplifying the effects of self-efficacy and risk perceptions, whereas benefit-driven pathways remain relatively stable across different LMX levels.

**Table 5:** LMX moderating effects.

Path	Low LMX	High LMX	Chi-square difference	*p*-value
SE → PR	−0.048	−0.970^***^	26.633	< 0.001
PR → IFS	−0.093	−0.361^***^	5.388	0.020
PR → DFS	−0.128	−0.458^***^	2.563	0.109
SE → PB	0.179^*^	0.509^***^	4.721	0.030
PB → DFS	0.585^***^	0.601^***^	0.019	0.890
PB → IFS	0.329^***^	0.372^***^	0.251	0.617

SE, perceived self-efficacy; PR, perceived risk; PB, perceived benefit; DFS, direct feedback seeking; IFS, indirect feedback seeking.

^***^p < 0.001 and ^*^p < 0.05.

## Discussion

The baseline structural equation model verified that academic self-efficacy significantly positively predicts perceived feedback benefit and significantly negatively predicts perceived feedback risk; perceived feedback benefit exerts a significant positive effect on both direct and indirect feedback seeking, while perceived feedback risk has a notable negative influence on the two types of feedback-seeking behaviors. Multi-group chi-square difference tests further examined the moderating effects of gender and Student-LMX. For gender, none of the model paths showed statistically significant group differences at the 5% significance level, indicating gender does not produce a prominent moderating effect on the hypothesized chained mediation relationships. By contrast, Student-LMX significantly moderates four core paths: the positive predictive path from self-efficacy to perceived benefit, as well as the negative predictive paths from self-efficacy to perceived risk and from perceived risk to direct and indirect feedback seeking. Specifically, the predictive strengths of these relationships are far stronger among students with high Student-LMX than those with low Student-LMX.

### Self efficacy and feedback seeking

A core finding of this study is that self-efficacy significantly predicts feedback seeking behavior among undergraduates. This finding is consistent with the meta-analytic conclusion of (Anseel et al. 2015), which confirmed that self-efficacy is positively associated with feedback seeking behavior.

Recent studies have refined the understanding of the self-efficacy–feedback seeking relationship, generally confirming a positive association. (Lu et al. 2022) found in a longitudinal study that higher academic self-efficacy mediated the link between learning goal orientation and subsequent feedback seeking, (Wei et al. 2022) reported that high-self-efficacy students were more active in seeking instructor advice, and Gan et al. (2021) showed that English language self-efficacy significantly influenced feedback behavior and preferences. However, this relationship is not uniformly positive, Xu and Wang (2024) found that linguistic self-efficacy negatively predicted feedback inquiry, whereas performance and self-regulatory self-efficacy positively predicted it. This suggests that the dimensional structure of self-efficacy may produce differential effects, calling for further research into how distinct self-efficacy dimensions shape feedback seeking processes (Cervone et al., 1991).

### The mediating role of cost–value perceptions

Another core finding of this study is that perceived benefit and perceived risk serve as dual mediators in the relationship between self-efficacy and feedback seeking. This result supports the cost-value framework of feedback seeking, which posits that individuals weigh the anticipated benefits of seeking feedback against its potential costs before deciding whether to initiate feedback seeking (Ashford and Cummings, 1983; Vandewalle, 2004; Sun, 2021).

In educational contexts, Spooner et al. (2024) found that perceived feedback benefit was positively associated with both feedback monitoring and inquiry. However, their quantitative data did not support a significant predictive role for perceived cost, even though personal cost emerged as a strong theme in interviews—a finding that contrasts interestingly with the present study, in which perceived risk significantly negatively predicted feedback seeking, albeit with relatively modest effect sizes. This discrepancy may reflect the inherent limitations of quantitative research in capturing social costs such as face loss, consistent with the observation of Young and Carless (2024) that although students recognize the potential benefits of seeking feedback, they nevertheless tend to avoid seeking it from instructors.

### Gender differences

This study did not find significant moderating effects of gender on the hypothesized pathways. This finding is consistent with (Spooner et al. 2024), who also observed no subgroup differences based on gender, study location, or student nationality. The systematic review by (Leenknecht and Carless 2023) similarly noted that although gender is frequently included as a control variable in feedback seeking research, evidence for its moderating role remains inconsistent.

One possible explanation is that the influence of gender roles on feedback seeking behavior is diminishing in contemporary higher education environments. With equalizing educational opportunities and converging learning contexts, male and female students may undergo similar cognitive processes when facing feedback seeking decisions, appraising self-efficacy, weighing costs and benefits, and perceiving the student–teacher relationship. Furthermore, in the Chinese higher education context in which this study was conducted, male and female students face similar opportunities and expectations with respect to academic engagement and student–teacher interaction, which may further attenuate gender-based differential effects.

### The moderating role of student-LMX

This study found that student-LMX significantly moderated the risk pathway (SE → PR → DFS/IFS) but did not significantly moderate the benefit pathway (SE → PB → DFS/IFS). This finding offers new insights into the role of student–teacher relationships in feedback seeking processes.

From the perspective of face culture in the Chinese context, feedback seeking is particularly challenging due to face concerns. Hwang et al. (2002) demonstrated that the “fear of losing face” (kiasuism), rooted in Chinese face culture, significantly shapes students' feedback seeking behaviors. Chen et al. (2023) further found that perceived face threat mediates the relationship between negative leadership and employee feedback seeking, suggesting that face concerns are a key psychological mechanism inhibiting proactive feedback seeking. High LMX signals that the teacher holds higher expectations and directs greater attention toward the student. Under these conditions, students with high self-efficacy become more invested in maintaining the relationship and more concerned about how they are perceived by the teacher. Consequently, they become sensitive to feedback that might reveal discrepancies between their actual performance and the teacher's expectations. What they perceive, then, is not the usefulness of feedback, but its potential to expose shortcomings. This cognitive process effectively rewires the perceived value of feedback into perceived risk—transforming what should be a developmental resource into a relational threat. Moreover, situated within a face-conscious cultural context, this tendency is further amplified, as the social cost of exposing inadequacies is considerably higher in collectivist and hierarchical settings where maintaining interpersonal harmony and social image is paramount. When students also possess high self-efficacy, their need to maintain face becomes even stronger-because they believe they ought to perform well, and any feedback that challenges this self-perception constitutes a greater face threat. Consequently, feedback seeking, which inherently entails admitting incompetence or dependence, is perceived not merely as a learning opportunity but as a potential source of social embarrassment.

Notably, the benefit pathway was not significantly moderated by LMX. This finding suggests that students' cognitive appraisal of the instrumental value of feedback is relatively independent of the quality of their relationship with the teacher—regardless of LMX level, students can rationally recognize that feedback is useful for performance improvement. This further suggests that the primary function of LMX is to “activate” the risk pathway rather than to “enhance” the benefit pathway.

### Implications

Cultivating students' self-efficacy is foundational to promoting feedback seeking. The present study found that self-efficacy promotes feedback seeking by enhancing perceived benefit and reducing perceived risk. Educators can enhance students' self-efficacy through providing mastery experiences, vicarious experiences, and verbal persuasion (Bandura et al., 1997), thereby strengthening students' intrinsic motivation to seek feedback. However, simply building good relationships with students or boosting their confidence is insufficient to reduce the social costs of feedback seeking. Rather, educators must explicitly cultivate a feedback culture in which seeking feedback is framed as a normative, growth-oriented practice rather than a sign of incompetence. In the Chinese cultural context, where face concerns are particularly salient, teachers should be attentive to face-saving practices—providing constructive feedback in private settings, normalizing feedback seeking as a routine academic activity, and clearly communicating that seeking feedback is encouraged and valued. By embedding these practices into the curriculum, educators can help students reinterpret feedback seeking as an opportunity for growth rather than a social threat, thereby transforming high-quality LMX and high self-efficacy into genuine buffers against perceived risk rather than unintended amplifiers of it.

### Limitation and future studies

This study has several limitations. First, the cross-sectional design precludes definitive causal inferences. Future research could employ longitudinal designs or cross-lagged panel designs to examine causal relationships among the variables. Second, the data relied entirely on self-report measures, raising the possibility of common method bias. Future research could employ multi-source data (e.g., combining instructor evaluations with student self-reports) to further mitigate method variance. Third, the sample was drawn exclusively from Chinese university students; the external validity of the findings awaits cross-cultural validation. Future research could test the theoretical model in different cultural contexts.

## Conclusion

Grounded in social cognitive theory and the cost-value framework, this study constructed and tested an integrated model examining how self-efficacy influences direct and indirect feedback seeking through perceived benefit and perceived risk, as well as the moderating roles of LMX and gender. The findings revealed that self-efficacy promotes feedback seeking by enhancing perceived benefit and reducing perceived risk; LMX significantly moderated the risk pathway but not the benefit pathway; and no significant moderating effect of gender was found. These findings illuminate the cognitive mechanisms and boundary conditions underlying the relationship between self-efficacy and feedback seeking, offering both theoretical contributions and practical implications for research on feedback seeking in educational contexts.

## Data Availability

The raw data supporting the conclusions of this article will be made available by the authors, without undue reservation.
